# A decade of public engagement regarding human germline gene editing: a systematic scoping review

**DOI:** 10.1038/s41431-024-01740-6

**Published:** 2024-11-28

**Authors:** Wendy P. Geuverink, Diewertje Houtman, Isabel R. A. Retel Helmrich, Joosje D. Kist, Lidewij Henneman, Martina C. Cornel, Sam R. Riedijk, ‘The DNA dialogues’ Consortium, Wendy P. Geuverink, Diewertje Houtman, Isabel R. A. Retel Helmrich, Joosje D. Kist, Lidewij Henneman, Martina C. Cornel, Sam R. Riedijk

**Affiliations:** 1 Amsterdam UMC, location Vrije Universiteit Amsterdam, Department of Human Genetics, Amsterdam, the Netherlands; 2 Amsterdam Reproduction and Development Research Institute, Amsterdam, the Netherlands; 3 Erasmus Medical Center, Department of Clinical Genetics, Rotterdam, the Netherlands; 4 Amsterdam Public Health Research Institute, Amsterdam, the Netherlands

**Keywords:** Ethics, Health policy, Genetic engineering

## Abstract

Following the discovery of the CRISPR-Cas technology in 2012, there has been a growing global call for public engagement regarding the potential use of human germline gene editing (HGGE). In this systematic scoping review, we aim to evaluate public engagement studies considering the following questions based on three points of attention: 1) Inclusion of underrepresented groups: who have been engaged? 2) Gathering values: what output has been reported? 3) Reaching societal impact: what objectives of public engagement have been reported? A systematic literature search from 2012 to 2023 identified 3464 articles reporting on public engagement studies regarding HGGE retrieved from 12 databases. After screening, 52 full-text articles were assessed for eligibility, resulting in 36 articles that cover 31 public engagement studies. We conclude that co-created efforts are needed to engage underrepresented groups as well as to yield values rather than acceptance levels, and to concretise how engagement might result in societal impact.

## Introduction

Following the discovery of the CRISPR-Cas technology in 2012, potential applications in somatic gene editing and human germline gene editing (HGGE) have received much attention. While somatic gene editing therapies using CRISPR-Cas9 have recently been introduced for clinical use [1, 2], the development and use of HGGE is considered more controversial [3]. Prohibitions are found in legislation in Europe and elsewhere [4–6]. Ethical questions about HGGE concern not only individuals and their offspring, but also society and humanity [7, 8].

While the call for public engagement regarding HGGE has intensified over the past decade, ideas on “why” and “how to” organize public engagement have evolved [9–15]. Societal alignment, i.e., aligning the goals and governance of science with the values in society, is increasingly considered the ultimate goal of public engagement efforts [11, 16–18]. The World Health Organization (WHO) formulates this as aligning “the research and policy agenda” with “public values, experiences, interests and priorities”. This has important implications for how to organize public engagement on HGGE. Building on the WHO framework for governance report on human genome editing we identified three points of attention in this regard: inclusion of underrepresented groups, gathering values, and reaching societal impact [11].

First, to align science with the values in society, it is necessary to gather the broad range of perspectives and underlying values present in society [19]. Public engagement therefore needs to be inclusive and should deliberately aim to gather perspectives of those who are typically underrepresented, such as groups with low trust in science [20, 21]. The WHO indicates that in addition to learning from the general public “specific strategies are needed to engage traditionally underrepresented groups, such as indigenous peoples, minority ethnic groups or faiths, or specific patient groups” [11]. This is important, because without representation of a broad diversity of perspectives, values, and types of knowledge, the development of societally aligned governance of HGGE will be threatened.

Second, to allow for societal alignment, public engagement should aim to yield an in-depth understanding of people’s values to align those with the research and policy agendas [11, 17, 22, 23]. In the literature, the term ‘values’ is used ambiguously; terms such as values, beliefs, moral considerations, concerns or attitudes are used interchangeably. Here we adhere to the Royal Commission on Environmental Pollution that defines values as “beliefs, either individual or social, about what is important in life, and thus about the ends or objectives which should govern and shape public policies” [24]. In addition, they report two characteristics of values: “they may be both formed and modified as a result of information and reflection” and “they emerge out of debate, discussion and challenge, as they encounter new facts insights and judgments contributed by others”. To collect those values, reflection and/or encounter with others is therefore important in public engagement.

Third, merely collecting values does not automatically feed into policy development [17]. For engagement practices to be consequential the steps after engagement towards societal alignment need to be specified as well. Public engagement practices should directly lead to output, i.e., insights that are the result of the practice. Examples can be insight into values of certain groups, or into strategies that are effective for engagement. Subsequently, these outputs can be used by stakeholders to change their behaviour, relationships, actions and activities, which can eventually lead to impact. For example, political parties can use the insights into underlying values to shape their government programme, after which citizens can vote for the party that best represents their perspective. In this way, public engagement can eventually lead to societal impact [25].

In this scoping review, we examine how calls for public engagement on HGGE have been put into practice from 2012 to this date. Ongoing developments over the past decade on why and how to organize public engagement may affect the extent to which the three points of attention mentioned (inclusion of underrepresented groups, gathering values, and reaching societal impact), have been part of the research design of the engagement studies reviewed in this paper. To enable a broader assessment of the engagement efforts, we respectively address the following research questions: 1) Who have been engaged? 2) What output has been reported? 3) What objectives of public engagement have been reported? The discussion interprets the results in light of the aforementioned points of attention.

## Methods

For this systematic scoping review, the PRISMA-ScR (Preferred Reporting Items for Systematic reviews and Meta-analysis extension for Scoping Reviews) guidelines, were followed [26].

### Search strategy

A systematic literature search was undertaken in December 2023 to identify articles that report on public engagement studies regarding HGGE. The search strategy was developed in consultation with a medical librarian. A detailed description is provided in Supplementary Information Text 1. Searches were performed in 12 databases, covering a range of disciplines: Medline, Embase, Web of Science, Scopus, Cinahl, Psychinfo, International Bibliography of the Social Sciences (IBSS), The Philosopher’s Index, Hein online, Google Scholar, Google, JSTOR. Two reviewers (D.H., W.G.) independently screened the titles and abstracts according to the inclusion and exclusion criteria (Supplementary Information Table 1). Only articles that were published after 2012 were included; after the discovery of CRISPR-Cas as a more precise and efficient gene editing technology [27]. Records were excluded if they were published before 2012, in a language other than English, if they concerned non-human or somatic gene editing and if focussed on experts, scientists, healthcare professionals and medical students. However, if the public engagement study included the latter groups alongside the public (including patients), they were included. In the current study, patients are considered a subgroup of the general public. In case of disagreement between the reviewers, consensus was established through discussion.

### Data extraction

A data-extraction table was developed. The two reviewers (W.G., D.H.) independently extracted the data from half of the articles, after which the extraction was checked by the second reviewer. The reviewers discussed the results and continuously updated the data-extraction table in an iterative process. The following information was summarized and used to answer the research questions (1): study participants (patients, public or both), year of publication, country of participants, sample size, sampling strategies and study limitations according to the authors (2), type of output (clustered in three categories), the methods, and type of research (qualitative or quantitative) (3), goal/objective of the study.

## Results

**Fig. 1:**
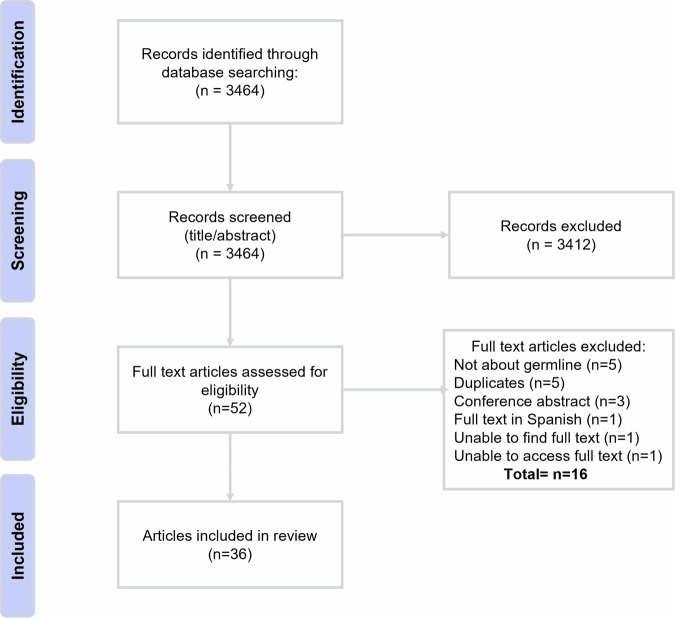
PRISMA-ScR flow diagram of article inclusion.

In total 3464 records were retrieved (Fig. 1). After screening, 52 full text articles were assessed for eligibility. In the full-text screening, 16 articles were excluded based on the exclusion criteria and clarified by the reasons recorded (Fig. 1), resulting in a total of 36 articles (Table 1). Three engagement studies were reported on two times [28–33], and one study was reported on three times answering different research questions [34–36]. These articles were considered the same engagement study, resulting in a total of 31 studies (Table 1).

**Table 1:** Characteristics of included articles.

Reference	First Author & Year of Publication	Methods	Participants*	Sample Size	Output(s)	Objective(s) of Public Engagement
**Patients**						
[58]	Hoffman-Andrews et al. [58]	Interviews	US; Adults with a diagnosis of Leber congenital amaurosis (LCA) or Retinitis Pigmentosa (RP)	*N* = 17	In-depth reasoning	Hearing a variety of voices
[37]	Hollister et al. [37]	Survey & Focus groups	US; Individuals (18 + ) with Sickle Cell Disease (SCD), parents of individuals with SCD	*N* = 87	Various outputs	Hearing a variety of voices
[30]	Snure Beckman et al. [30]^a^	Interviews	US; Parents of living or deceased children diagnosed with full or mosaic trisomy 21, 18, or 13	*N* = 27	In-depth reasoning	Hearing a variety of voices
[31]	Elliott et al. [31]^b^	Interviews	US; Parents of living or deceased children diagnosed with full or mosaic trisomy 21, 18, or 13	*N* = 27	In-depth reasoning	Hearing a variety of voices/ Informing policy making
[60]	Van Dijke et al. [60]	Interviews	The Netherlands; High-risk couples (different inheritance patterns and having a living or deceased child with a genetic condition) or one of the parents being a carrier of an inherited autosomal dominant disorder.	*N* = 25	In-depth reasoning	Hearing a variety of voices/ Informing policy making
[61]	Geuverink et al. [61]	Interviews	The Netherlands; Carriers of various inherited autosomal dominant disorders	*N* = 10	In-depth reasoning	Hearing a variety of voices
[59]	Neuhausser et al. [59]	Survey	US; Patients at a university affiliated infertility practice	*N* = 172 (in 2021) & 469 (in 2018)	Level of acceptance	Insight into public attitude forming
**Public**						
[29]	McCaughey et al. [29]^a^	Survey	185 countries	*N* = 12,562	Various outputs	Insight into public attitude forming
[28]	McCaughey et al. [28]^b^	Survey	185 countries	*N* = 3935	Various outputs	Insight into public attitude forming/ Monitor or increase public knowledge or support/ Informing policy making
[36]	Scheufele et al. [36]^a^	Survey	US	*N* = 1600	Level of acceptance	Insight into public attitude forming
[35]	Howell et al. [35]^b^	Survey	US	*N* = 1484	Level of acceptance	Insight into public attitude forming
[34]	Eichmeier et al. [34]^c^	Survey	US	*N* = 1600	Level of acceptance	Insight into public attitude forming
[55]	Gaskell et al. [55]	Survey	Austria, Denmark, Germany, Hungary, Iceland, Italy, the Netherlands, Portugal, Spain, UK (EEA-10 countries) & the United States	*N* = 11,716	Various outputs	Monitor or increase public knowledge or support/ Informing policy making
[41]	Weisberg et al. [41]	Survey	US	*N* = 2493	Level of acceptance	Insight into public attitude forming/ Monitor or increase public knowledge or support/ Informing policy making
[47]	Hendriks et al. [47]	Survey	The Netherlands	*N* = 1013	Various outputs	Insight into public attitude forming
[50]	Treleaven and Tuch [50]	Focus groups	Australia	*N* = 46	Level of acceptance	Informing policy making
[51]	Critchley et al. [51]	Survey	Australia	*N* = 1004	Level of acceptance	Insight into public attitude forming
[42]	Riggan et al. [42]	Focus groups	US	*N* = 50	In-depth reasoning	Hearing a variety of voices/ Insight into public attitude forming/ Informing policy making
[38]	Kaur [38]	Survey	UK	*N* = 521	Various outputs	Hearing a variety of voices
[56]	Jedwab et al. [56]	Survey	67 countries; Majority from US, Australia, Canada, UK,	*N* = 1537	Level of acceptance	Hearing a variety of voices/ Insight into public attitude forming/ Informing policy making
[39]	Schuijff et al. [39]	Survey & Focus groups	The Netherlands	*N* = 30	Various outputs	Insight into public attitude forming/ Informing policy making
[48]	Van Dijke et al. [48]	Survey	The Netherlands	*N* = 1136	Level of acceptance	Insight into public attitude forming/ Informing policy making
[54]	So et al. [54]	Survey	US & Canada	*N* = 400	Various outputs	Insight into public attitude forming
[43]	McFadden et al. [43]	Focus groups	US	*N* = 64	In-depth reasoning	Insight into public attitude forming
[44]	Chen and Zhang [44]	Survey	US	*N* = 2105	Level of acceptance	Insight into public attitude forming
[57]	Busch et al. [57]	Survey	Canada, US, Austria, Germany, Italy	*N* = 3698	Level of acceptance	Insight into public attitude forming/ Informing policy making
[49]	Houtman et al. [49]	Survey	The Netherlands	*N* = 2795	Various outputs	Hearing a variety of voices/ Informing policy making
[52]	Thaldar et al. [52]	Survey & Deliberations	South Africa	*N* = 29	Various outputs	Informing policy making
[32]	Akatsuka et al. [32]^a^	Survey	Japan	*N* = 4424	Level of acceptance	Hearing a variety of voices
[33]	Sawai et al. [33]^b^	Survey	Japan	*N* = 4424	Level of acceptance	Hearing a variety of voices/ Insight into public attitude forming
[45]	Halstead et al. [45]	Survey	US	*N* = 4726	Level of acceptance	Insight into public attitude forming/ Informing policy making
[53]	Macall et al. [53]	Survey	Costa Rica	*N* = 1096	Level of acceptance	Hearing a variety of voices
[40]	Jibrilla et al. [40]	Survey	Nigeria	*N* = 188	Level of acceptance	Informing policy making
[46]	Nelson et al. [46]	Focus groups	US	*N* = 32	In-depth reasoning	Hearing a variety of voices/ Informing policy making
**Public & Patients**						
[62]	Uchiyama et al. [62]	Survey	Japan; General public & adult patients who indicated that their disease conditions were related to their “genetic makeup”	*N* = 10,881 (general public) N = 1044 (patients)	Level of acceptance	Hearing a variety of voices/ Insight into public attitude forming
[63]	Van Baalen et al. [63]	Dialogues	The Netherlands; General public & (child) patients and their family members	*N* = 804 (general public) *N* = 20 ((child) patients and family)	In-depth reasoning	Hearing a variety of voices/ Informing policy making

* “experts” not included.

^abc^Engagement studies based on the same sample: Three engagement studies were reported on two times, and one engagement study was reported on three times answering different research questions.

**Fig. 2:**
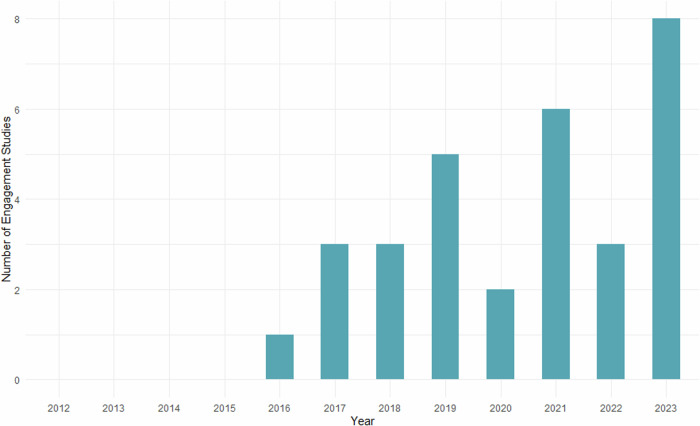
The number of engagement studies regarding HGGE per year since 2012 (*N* = 31).

Figure 2 shows the number of studies on public engagement regarding HGGE published since 2012. We did not find any scientific publications that were published between 2012 and 2015. From 2016 onwards, there has been a steady increase in the number of studies.

### Who have been engaged?

**Fig. 3:**
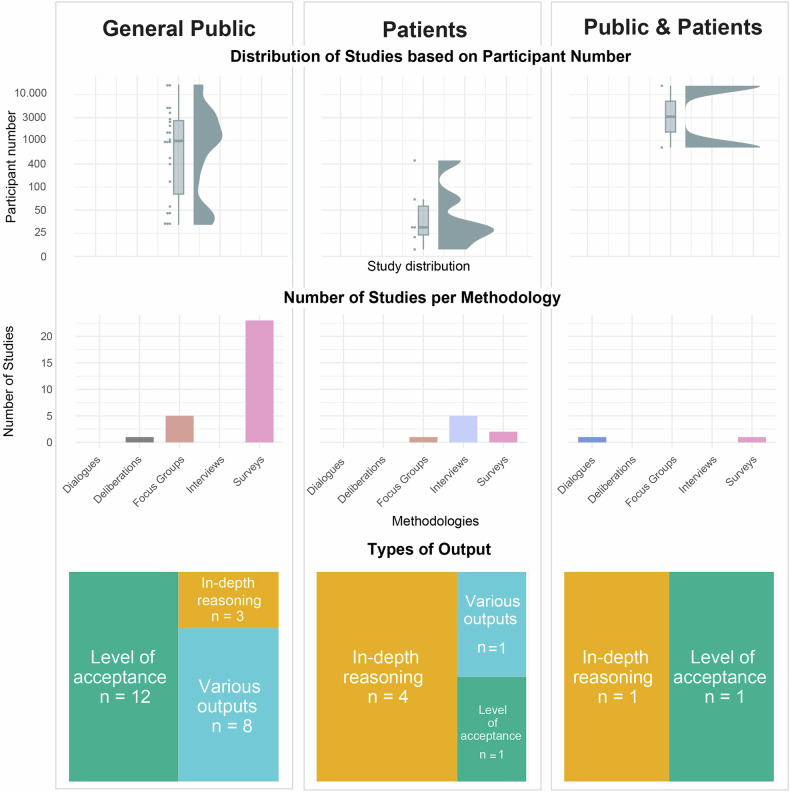
The total number of participants, the number of studies per methodology and the types of output for each participant category (general public, patients and public & patients).

Of the 31 studies identified, 23 studies (74%) described their participants as general public, six studies (19%) focussed on patients and two studies (6%) included both patients and general public (Fig. 3). Five studies had professional experts (e.g., scientists, healthcare professionals) or (medical) students participating besides the general public and/or patients [32, 33, 37–40].

From the 23 studies focussing on the general public, there were seven studies engaging participants from the US [34–36, 41–46], four from the Netherlands [39, 47–49], two from Australia [50, 51], one from South Africa [52], one from Costa Rica [53], one from the US and Canada combined [54], one from the UK [38], one from Japan [32, 33] and one from Nigeria [40], and four studies engaged participants ranging from 5 to 185 different countries [28, 29, 55–57] (Table 1).

Out of the six studies focussing on patients, four engaged patients from the US [30, 31, 37, 58, 59] and two engaged patients from the Netherlands [60, 61] (Table 1). Patient populations included (parents of) people with Retinitis Pigmentosa, Leber Congenital Amaurosis [58], Sickle Cell Disease [37], chromosomal conditions (trisomy 21,18,13) [30, 31], or various autosomal dominant disorders (e.g., Huntington’s disease, Hereditary breast and ovarian cancer, Myotonic Dystrophy Type 1) [61], couples at increased risk of having a child with a genetic disorder (e.g., Hereditary breast and ovarian cancer, cystic fibrosis, Fragile-X syndrome [60], and patients with fertility problems attending an infertility clinic [59]. Two studies that focussed on the general public also specifically asked about genetic conditions, thereby identifying a subgroup of patients (and their families) among their participants [29, 38].

Out of the two studies where both the general public and patients participated, one engaged participants from Japan [62], and one from the Netherlands [63]. Patients who participated in these studies included individuals diagnosed with or at risk of developing a variety of genetic conditions [62] and child- and adult patients and their family members [63].

Who have been engaged was also influenced by various sampling strategies aiming to increase inclusivity, diversity and representativeness. For example, one study used tailored recruitment and dialogue strategies for specific groups, such as children and adolescents, people with low literacy and citizens with a migrant background [63]. Another study selected general public participants based on sex, age, and residential area according to the national census data [62]. More strategies can be found in the Supplementary Information Text 2.

Despite these sampling strategies, 27 studies (87%) reported biases in their sample [28–38, 40, 41, 44–62]. For example, they included more participants who are highly educated, and predominantly white [41, 56]. Nineteen studies used online engagement methods (e.g., online surveys or online focus groups), thereby excluding people who do not have internet access [28, 29, 32–36, 38, 41, 44–49, 51–57, 62]. The studies including patients and family of patients also indicated that participants’ representativeness of the targeted patient population was limited. For example, because participants were members of a community/advocacy/condition-specific support group [30, 31, 37, 58]. For more details, see Supplementary Information Text 3.

### What output has been reported?

In terms of output, 14 studies (45%) focussed on level of acceptance, support, or agreement regarding one or multiple potential applications of HGGE (Table 1 and Fig. 3) [32–36, 40, 41, 44, 45, 48, 50, 51, 53, 56, 57, 59, 62]. The majority of these studies also investigated associations between acceptance and other variables [34, 35, 41, 44, 48, 51, 56, 59, 62], for example professional, personal or family related experiences with genetics or genomics [56]. One study investigated the degree to which attitudinal profiles could be distinguished [45] (Supplementary Information Text 4).

Nine of the 31 engagement studies (29%) yielded various outputs [28, 29, 37–39, 47, 49, 52, 54, 55] In these studies, the level of acceptance or support was complemented with more in-depth reasoning. In half of the studies [28, 29, 47, 52, 54, 55] there was a combination of a survey with both a focus on the acceptability of different HGGE and/or somatic gene editing scenarios, followed by one or more open-ended questions asking for a qualitative reasoning or justification of their choices, or assessing the determinants of the participants’ opinions. The other half of the studies [37–39, 49] yielded both qualitative and quantitative outputs. For example, in one study, participants indicated their moral acceptance level on a pre- and post-video survey in combination with a more in-depth exploration of values through focus groups [37] (Supplementary Information Text 4).

A further eight of the 31 engagement studies (26%) focused on in-depth reasoning regarding the potential use of HGGE research and applications [30, 31, 42, 43, 46, 58, 60, 61, 63] Within these studies, rather than focusing on levels of acceptance or support, an in-depth exploration of attitudes, feelings, thoughts, values, perspectives, moral considerations, lived experiences, hopes, wishes, concerns, expectations, conditions and priorities was reported.

Although all engagement studies aimed to capture the perspectives of participants, authors also reported limitations that may affect the validity of their outputs [28–35, 37, 41, 42, 45, 47, 48, 50, 52, 56, 57, 59, 60, 63] For example: respondents were asked to assume that HGGE was safe and effective [56] not all characteristics of HGGE were included to limit complexity of the information provided [48] texts were kept short which may have led to uninformed answers [57] or examples of HGGE applications were not included which may have made it difficult to form an opinion [32, 33] (Supplementary Information Text 5).

The reported output was generated by various methods. The majority of studies (19; 61%) used (online) surveys [28, 29, 32–36, 38, 40, 41, 44, 45, 47–49, 51, 53–57, 59, 62] (Table 1 and Fig. 3). Four studies (13%) conducted semi-structured interviews [30, 31, 58, 60, 61] Four studies (13%) reported on focus groups [42, 43, 46, 50] One study (3%) organized dialogues [63] and three studies (10%) used multiple methods, where one study used Q-methodology along with focus groups [39] one study used online voting polls in combination with online group deliberations [52] and one study used a survey in combination with focus groups [37].

### What objectives of public engagement have been reported?

All studies aimed to capture the perspectives of the general public and/or a specific (patient) group. Various studies also aim to investigate associations between variables and participants’ perspectives on HGGE [28, 29, 33–36, 39, 41–45, 47, 48, 51, 54, 56, 57, 59, 62] Moreover, the majority of included studies reported on the objectives of public engagement beyond their study, indicating that the results of the studies are important to inform policy-making, revise legislation and democratize science development [28, 31, 36, 39–42, 45, 46, 48–50, 52, 55–57, 60, 61, 63] (Supplementary Information Text 6). For example, in one study a call for urgent revision of ethics guidelines and for dedicated HGGE legal regulations was made based on the public opinions and yielded reasons behind it that emerged from their engagement practice [52] Another study mentioned that “users’ perspectives should be addressed, and they should be involved in shared governance and guiding further science and policy-making” [60] Although these studies all indicate that public engagement should inform policy-making, none of these studies describe in what way the results of their engagement practice should feed into policy development.

## Discussion

This article aimed to systematically review how calls for public engagement on HGGE have been put into practice from 2012 to December 2023. From 2016 onwards there is an increasing trend in publications. We identified 31 engagement studies reported on in 36 articles. Although calls for public engagement on HGGE have been made in recent years, we noticed a contrast between the hundreds of perspectives and opinion articles on HGGE, compared to only 31 studies in which the public was actually engaged about HGGE. Moreover, the engagement studies were disproportionately concentrated in countries of the Global North. Here we answer the research questions and discuss the results in light of the identified three points of attention: inclusion of underrepresented groups, gathering values, and reaching societal impact [11].

First, the majority of engagement studies on HGGE focused on the general public. In addition, a substantial number of studies engaged patients. Both the US and the Netherlands stand out as the two countries from which we identified the most published engagement practices on HGGE, namely, 11 and 7 studies, respectively. The WHO indicates that the public dialogue on HGGE has to be conducted nationally, given the various historical, cultural and religious contexts, but also stresses the need for a global approach when it comes to governance measures [11] This raises important questions, such as how to involve everyone with a stake in the human genome [15] Continued attention to these profound questions is needed to ensure ethical values and principles such as social justice, solidarity and global health justice that underpin decisions made [11] This includes for example “A commitment to equitable access to opportunities and potentially beneficial outcomes from human genome editing for all people, particularly those living in low- and middle-income countries” [11] On a study level, multiple sampling strategies were described to increase inclusivity, diversity and representativeness. Most studies mainly focused on efforts to increase demographic representativeness by trying to create a representative sample that reflects the characteristics of the population. In most studies these efforts did not lead to representative samples. Moreover, these strategies do not necessarily lead to the inclusion of underrepresented groups. Without the use of specific strategies that are tailored to the motives and needs of certain target groups it is likely that primarily those who are already interested in science and those who trust science, will be reached [64] Without acknowledging that motives for public engagement may not be shared by certain groups, and that some people may experience barriers such as sensitivities towards the subject, these people will be (unintentionally) excluded [20] Although in charge of recruiting participants in public engagement, researchers and other initiators often do not reduce these barriers or address the needs and concerns of these target groups themselves. When it comes to efforts to include underrepresented groups in this review, we found that only one study tailored dialogues to reach specific target audiences [63] By building contacts with key figures who originate from or have the trust of certain underrepresented groups, these groups might be more equally involved [65] Efforts to include specific groups (not necessarily underrepresented) are seen in the patient studies. Patients bring different experiences for example because they have a (child with a) genetic disorder and/or they are at risk of having affected offspring [66] If HGGE would become available, these patient groups could also be (family members of) the future potential users. Based on their lived experience and knowledge, they can share which values are at stake and might govern and shape policymaking. Overall, many efforts to include a representative, diverse or inclusive sample were reported, however the success seems limited and there is little focus on identifying or engaging underrepresented groups. Thus, we conclude that in future engagement practices more co-created efforts are needed to engage underrepresented groups (Box 1; recommendation 1).

Second, although the literature on public engagement in HGGE considers collecting of ‘values’ as crucial output [3, 11, 17–19] nearly half of the engagement studies focused on level of acceptance, support, or agreement regarding one or multiple potential applications of HGGE. This focus on acceptance levels does not reveal the underlying values based on which people consider certain applications acceptable or not. Questions about the extent to which people find certain specific uses of HGGE acceptable or not, do not adequately reflect the current reality in which HGGE is not safe, not effective and prohibited, nor is it clear whether these will ever become realistic scenarios. While asking for acceptance of certain uses may seem closer to “the goals or objectives that should define and shape public policy”, translation to policy remains challenging as long as proposed scenarios are far from reality and underlying values and reasons remain unknown. For example, asking participants to indicate their level of acceptance of HGGE with regard to severe genetic diseases leaves several questions unanswered, such as “What are severe genetic diseases?” and “What are the underlying values as to why this is considered more or less (un)acceptable?”. Moreover, exploring how to proceed with HGGE within the normative frameworks of the public engagement initiators leaves little room for participants to bring their values and needs into the conversation [9, 15, 18] As Martani [67] recently stressed: “one could say that the feature of broadness should thus concern not only the question as to ‘who is sitting at the debate table’, but also ‘what matters are placed, misplaced or not placed at all on said table’“ [67] In addition, for the majority of the studies the public engagement effort was conducted through an (online) survey. A survey is not the most suitable method to gain insight into people’s underlying values given the two main characteristics of values, namely that they are shaped by information and reflection and that they emerge from discussion and hearing new insights from others [24] Presenting possible future scenarios to participants could help to open up the conversation and leave room for different perspectives, including dissenting voices (e.g. that would wish to ban or limit research) [68] Methods that involve interaction between participants, such as dialogues, deliberations and focus groups or methods, such as interviews, that provide opportunities for probing and reflection are more likely to enable the evolvement, awareness and communication of values (Box 1; recommendation 2). Clearly, surveys allow for larger sample sizes than dialogues or interviews. There remains a trade-off between reaching many people and gaining an in-depth understanding of participants perspectives [64] This underlines the need to carefully consider the goals and desired output of public engagement and to align the methods with these goals (Box 1; recommendation 3).

Third, although the majority of included studies indicate the importance of public engagement for informing policy, none of these studies specify how the results of engagement practice should inform policy development and should contribute to societal alignment. Here it is important to note that best practices for public engagement and its desired consequences have evolved over the past decade and are still under development [9–15, 18] These practices increasingly take place in a transdisciplinary context where insights regarding gene editing technology, combined with other relevant disciplines such as science communication, sociology, science and technology studies, and governance need to be taken into account in shaping public engagement. This transdisciplinary collaboration requires competencies of which the importance is increasingly recognized and for which the field is currently building capacities. If informing policy-making, reviewing legislation and democratizing scientific development is the objective of a study, it is important to be aware of the power associated with the task of interpreting the views of the public. For two reasons, interpretations of findings from public engagement activities may not accurately reflect participants’ views and values. First, there is a risk that the results may be presented in terms of common morality, removing the nuance and conflicting values that were present in the public engagement [18] Second, if the interpreters are too homogeneous or have conflicts of interest, there is a risk of technocracy or propaganda [18, 69] To mitigate these interpretation risks, it is important to test whether participants recognize their views in the interpretation and to assign the interpretation task to an independent group of people in which a variety of perspectives are represented. (Box 1; recommendation 4). Moreover, research does not automatically lead to societal impact. It requires a detailed plan for how results will feed towards impact; who needs to use the research insights and in what way, to reach the impact that is envisioned [27] thereby enabling internal and external communication and evaluation. The studies we included may have worked with such theories and planned activities for societal alignment, however, it is not common practice to describe this in scientific publications. In addition, while the pursuit of societal alignment encourages the acknowledgement of differences in values of diverse actors which is considered important for the democratization of science, it is also described as a dilemma given the difficulties of decision-making in the context of these various and possibly conflicting values [16] The Oviedo Convention re-examination of Article 13, is an example where the output of public engagement efforts, including in-depth dialogues among a wide range of stakeholders on a European level, was directly incorporated into the refinement of the ethical guidelines and regulatory frameworks [4] However, these examples are scarce and actually integrating a variety of perspectives into the policy making process remains a challenge [16, 64] Caution is needed when making policy recommendations regarding disruptive technologies such as HGGE. There is a risk that too much power resides with non-democratically elected institutions [18] Instead of advising the government based on public engagement findings, it may be better to inform rather than advise. Politicians and policymakers are then able to make informed decisions in line with their political preferences (Box 1; recommendation 5). Based on our results, we provide recommendations for public engagement regarding HGGE (Box 1).

Box 1 Recommendations for Public Engagement Practices 1. Co-create engagement methods for underrepresented groups

2. Use methods that enable interaction and reflection to yield values

3. Align goals, method and output of public engagement

4. Collaborate in a diverse and independent transdisciplinary consortium

5. Discuss and concretise how public engagement might produce societal impact

### Strengths and limitations

To our knowledge, this is the first review that systematically looks at methodology rather than content, i.e., perspectives regarding HGGE. Our systematic search of the literature was conducted by an experienced medical librarian. We searched for “germline” in the full text, in addition to the standard search focusing on search words in the title and abstract, in order to be as thorough as possible. In choosing to focus on peer-reviewed published articles regarding public engagement on HGGE in English only, we might have missed public engagement practices in other languages, those that are not published in the academic literature, or those that can only be found in grey literature. For example, public engagement practices conducted by advocacy, policy or private organizations such as ARRIGE (Association for Responsible Research and Innovation in Genome Editing) were missed [70] In reviewing the included studies, we categorized or clustered various results (output types and objectives), according to our interpretation. Other researchers may come to different categories. Despite these limitations, we believe our review has provided a clear overview of the methodological issues regarding public engagement efforts when it comes to answering the call for public engagement regarding HGGE.

## Conclusion

We conclude that 1) there is an increase of public engagement efforts since the discovery of CRISPR-Cas technology in 2012, 2) more co-created efforts are needed to engage underrepresented groups on a national level, 3) a focus on acceptance levels does not reveal the underlying values that should inform policy-making, and 4) translation from collected values to societal impact receives little attention in practice. This systematic scoping review shows that researchers around the world have embarked on the endeavor of public engagement regarding HGGE and are learning more about the practical challenges that need to be addressed. At the same time, there are still profound ethical challenges that deserve the utmost attention, about the human genome as humanity’s common property and what that means for who should be involved and in charge at national and global levels.

## Supplementary information

Supplementary Information

## References

[1] Wong C. UK first to approve CRISPR treatment for diseases: what you need to know. Nature. 2023;623:676–7. 37974039 10.1038/d41586-023-03590-6

[2] FDA Approves First Gene Therapies to Treat Patients with Sickle Cell Disease: FDA; 2023 [Available from: https://www.fda.gov/news-events/press-announcements/fda-approves-first-gene-therapies-treat-patients-sickle-cell-disease.

[3] Almeida M, Ranisch R. Beyond safety: mapping the ethical debate on heritable genome editing interventions. Humanities Soc Sci Commun. 2022;9:1–14.

[4] Genome editing technologies: final conclusions of the re-examination of Article 13 of the Oviedo Convention: Council of Europe; 2022 [Available from: https://www.coe.int/en/web/bioethics/-/genome-editing-technologies-final-conclusions-of-the-re-examination-of-article-13-of-the-oviedo-convention.

[5] Isasi R, Kleiderman E, Knoppers BM. Editing policy to fit the genome? Science. 2016;351:337–9. 26797999 10.1126/science.aad6778

[6] Embryowet: Overheid; 2021 [Available from: https://wetten.overheid.nl/BWBR0013797/2021-07-01.

[7] International Bioethics Committee. Report of the IBC on updating its reflection on the Human Genome and Human Rights. Paris: United Nations Educational, Scientific and Cultural Organization; 2015

[8] Council of Europe. Explanatory Report to the Convention for the protection of Human Rights and Dignity of the Human Being with regard to the Application of Biology and Medicine: Convention on Human Rights and Biomedicine: Council of Europe; 1997

[9] Andorno R, Baylis F, Darnovsky M, Dickenson D, Haker H, Hasson K, et al. Geneva Statement on Heritable Human Genome Editing: The Need for Course Correction. Trends in Biotechnology. 2020 10.1016/j.tibtech.2019.12.022 32014274

[10] Lander ES, Baylis F, Zhang F, Charpentier E, Berg P, Bourgain C, et al. Adopt a moratorium on heritable genome editing. Nature. 2019;567:165–8. 30867611 10.1038/d41586-019-00726-5

[11] WHO Expert Advisory Committee on Developing Global Standards for Governance and Oversight of Human Genome Editing. Human Genome Editing: recommendations. Geneva: World Health Organization, Governance HE; 2021. Contract No.: CC BY-NC-SA 3.0 IGO

[12] de Wert G, Pennings G, Clarke A, Eichenlaub-Ritter U, van El CG, Forzano F, et al. Human germline gene editing: Recommendations of ESHG and ESHRE. Eur J Hum Genet: EJHG. 2018;26:445–9. 29326428 10.1038/s41431-017-0076-0 PMC5891496

[13] Nuffield Council on Bioethics. Genome Editing and Human Reproduction: social and ethical issues. London; 2018

[14] National Academy of Medicine, National Academy of Sciences, and the Royal Society. Heritable Human Genome Editing. Washington, DC; 2020 32897669

[15] Jasanoff S, Hurlbut JB, Saha K. CRISPR democracy: Gene editing and the need for inclusive deliberation. Issues Sci Technol. 2015;32:37.

[16] Ribeiro B, Bengtsson L, Benneworth P, Bührer S, Castro-Martínez E, Hansen M, et al. Introducing the dilemma of societal alignment for inclusive and responsible research and innovation. J responsible Innov. 2018;5:316–31.

[17] Nelson JP, Selin CL, Scott CT. Toward anticipatory governance of human genome editing: A critical review of scholarly governance discourse. J Responsible Innov. 2021;8:382–420. 35281674 10.1080/23299460.2021.1957579 PMC8916747

[18] Evans JH. Can the public express their views or say no through public engagement? Environ Commun. 2020;14:881–5.

[19] Iltis AS, Hoover S, Matthews KRW. Public and Stakeholder Engagement in Developing Human Heritable Genome Editing Policies: What Does it Mean and What Should it Mean? Front Political Sci. 2021;3:730869.

[20] Geuverink WP, Houtman D, Retel Helmrich IRA, van Baalen S, van Beers BC, van El CG, et al. The need to set explicit goals for human germline gene editing public dialogues. J Community Genet. 2024;15:1–7. 10.1007/s12687-024-00710-1 PMC11217238 38720104

[21] Gunn C, Jongsma K. Inclusion by invitation only? Public engagement beyond deliberation in the governance of innovative biotechnology. Am J Bioeth. 2023;23:79–82. 38010683 10.1080/15265161.2023.2272930

[22] Sarewitz D. CRISPR: Science can’t solve it. Nature. 2015;522:413–4. 26108836 10.1038/522413a

[23] Salisbury J, Nicholas B. Review of public engagement in the development and oversight of emerging technologies (‘science and society’). Working paper prepared for the Lockhart review on human cloning and embryo. 2005

[24] Royal Commission on Environmental Pollution. Setting Environmental Standards. 1998

[25] RESOURCES FOR RESEARCH PLANNING AND EVALUATION: Sustainability Research Effectiveness; [Available from: https://researcheffectiveness.ca/resources-for-research-planning-and-evaluation/.

[26] Tricco AC, Lillie E, Zarin W, O’Brien KK, Colquhoun H, Levac D, et al. PRISMA extension for scoping reviews (PRISMA-ScR): checklist and explanation. Ann Intern Med. 2018;169:467–73. 30178033 10.7326/M18-0850

[27] Doudna JA, Charpentier E. The new frontier of genome engineering with CRISPR-Cas9. Science. 2014;346:1258096. 25430774 10.1126/science.1258096

[28] McCaughey T, Budden DM, Sanfilippo PG, Gooden GEC, Fan L, Fenwick E, et al. A need for better understanding is the major determinant for public perceptions of human gene editing. Hum gene Ther. 2019;30:36–43. 29926763 10.1089/hum.2018.033

[29] McCaughey T, Sanfilippo PG, Gooden GEC, Budden DM, Fan L, Fenwick E, et al. A global social media survey of attitudes to human genome editing. cell stem cell. 2016;18:569–72. 27152441 10.1016/j.stem.2016.04.011

[30] Snure Beckman E, Deuitch N, Michie M, Allyse MA, Riggan KA, Ormond KE. Attitudes toward hypothetical uses of gene-editing technologies in parents of people with autosomal aneuploidies. CRISPR J. 2019;2:324–30. 31599684 10.1089/crispr.2019.0021

[31] Elliott K, Ahlawat N, Beckman ES, Ormond KE. “I wouldn’t want anything that would change who he is.” The relationship between perceptions of identity and attitudes towards hypothetical gene-editing in parents of children with autosomal aneuploidies. SSM-Qualitative Res Health. 2022;2:100151.

[32] Akatsuka K, Hatta T, Sawai T, Fujita M. Genome editing of human embryos for research purposes: Japanese lay and expert attitudes. Front Genet. 2023;14:1205067. 37424733 10.3389/fgene.2023.1205067 PMC10324961

[33] Sawai T, Hatta T, Akatsuka K, Fujita M. Human genome editing in clinical applications: Japanese lay and expert attitudes. Front Genet. 2023;14:1205092. 37662845 10.3389/fgene.2023.1205092 PMC10469609

[34] Eichmeier AA, Bao L, Xenos MA, Brossard D, Scheufele DA. Fictional scenarios, real concerns: science fiction and perceptions of human genome editing. J Sci Commun. 2023;22:A08.

[35] Howell EL, Kohl P, Scheufele DA, Clifford S, Shao A, Xenos MA, et al. Enhanced threat or therapeutic benefit? Risk and benefit perceptions of human gene editing by purpose and heritability of edits. J Risk Res. 2022;25:139–55.

[36] Scheufele DA, Xenos MA, Howell EL, Rose KM, Brossard D, Hardy BW. US attitudes on human genome editing. Science. 2017;357:553–4. 28798120 10.1126/science.aan3708

[37] Hollister BM, Gatter MC, Abdallah KE, Armsby AJ, Buscetta AJ, Byeon YJJ, et al. Perspectives of sickle cell disease stakeholders on heritable genome editing. CRISPR J. 2019;2:441–9. 31742431 10.1089/crispr.2019.0034 PMC6919256

[38] Kaur A. Could seeking human germline genome editing force journeys of transnational care? Multidiscip J Gend Stud. 2020;9:184–209.

[39] Schuijff M, De Jong MDT, Dijkstra AM. AQ methodology study on divergent perspectives on CRISPR-Cas9 in the Netherlands. BMC Med Ethics. 2021;22:48. 33902573 10.1186/s12910-021-00615-5 PMC8074506

[40] Jibrilla M, Raji H, Okeke MI. Survey of attitude to human genome modification in Nigeria. J Community Genet. 2024;15:1–11. 37995060 10.1007/s12687-023-00689-1 PMC10857991

[41] Weisberg SM, Badgio D, Chatterjee A. A CRISPR new world: attitudes in the public toward innovations in human genetic modification. Front public health. 2017;5:253896. 10.3389/fpubh.2017.00117 PMC5439143 28589120

[42] Riggan KA, Sharp RR, Allyse M. Where will we draw the line? Public opinions of human gene editing. Qualitative Health Res. 2019;29:1823–35. 10.1177/1049732319846867 31057062

[43] McFadden BR, Rumble JN, Stofer KA, Folta KM, Turner S, Pollack A. Gene editing isn’t just about food: comments from US focus groups. GM Crops Food. 2021;12:616–26. 34014805 10.1080/21645698.2021.1919485 PMC9208619

[44] Chen AA, Zhang X. Rethinking the knowledge-attitudes model and introducing belief in human evolution: examining antecedents of public acceptability of human gene editing. Health, Risk Soc. 2022;24:297–316.

[45] Halstead IN, Boehnke JR, Lewis GJ. Heterogeneous attitudinal profiles towards gene editing: Evidence from latent class analysis. Public Underst Sci. 2023;32:159–74. 36003037 10.1177/09636625221114608

[46] Nelson JP, Tomblin DC, Barbera A, Smallwood M. The divide so wide: Public perspectives on the role of human genome editing in the US healthcare system. Public Underst Sci. 2024;33:189–209. 37638525 10.1177/09636625231189955

[47] Hendriks S, Giesbertz NAA, Bredenoord AL, Repping S. Reasons for being in favour of or against genome modification: a survey of the Dutch general public. Hum Reprod Open. 2018;2018:hoy008. 30895249 10.1093/hropen/hoy008 PMC6276646

[48] Van Dijke I, van Wely M, Berkman BE, Bredenoord AL, Henneman L, Vliegenthart R, et al. Should germline genome editing be allowed? The effect of treatment characteristics on public acceptability. Hum Reprod. 2021;36:465–78. 33242333 10.1093/humrep/deaa212 PMC8453417

[49] Houtman D, Vijlbrief B, Polak M, Pot J, Verhoef P, Cornel M, et al. Changes in opinions about human germline gene editing as a result of the Dutch DNA-dialogue project. Eur J Hum Genet. 2022:1-8 10.1038/s41431-022-01114-w PMC9095815 35551502

[50] Treleaven T, Tuch BE. Australian public attitudes on gene editing of the human embryo. J Law Med. 2018;26:204–7. 30302982

[51] Critchley C, Nicol D, Bruce G, Walshe J, Treleaven T, Tuch B. Predicting public attitudes toward gene editing of germlines: the impact of moral and hereditary concern in human and animal applications. Front Genet. 2019;9:704. 30687386 10.3389/fgene.2018.00704 PMC6334182

[52] Thaldar D, Shozi B, Steytler M, Hendry G, Botes M, Mnyandu N, et al. A deliberative public engagement study on heritable human genome editing among South Africans: Study results. Plos one. 2022;17:e0275372. 36441783 10.1371/journal.pone.0275372 PMC9704621

[53] Macall DM, Madrigal-Pana J, Smyth SJ, Arias AG. Costa Rican consumer perceptions of gene-editing. Heliyon. 2023;9:e19173. 10.1016/j.heliyon.2023.e19173 PMC10468379 37664745

[54] So D, Sladek R, Joly Y. Assessing public opinions on the likelihood and permissibility of gene editing through construal level theory. N. Genet Soc. 2021;40:473–97.

[55] Gaskell G, Bard I, Allansdottir A, Da Cunha RV, Eduard P, Hampel J, et al. Public views on gene editing and its uses. Nat Biotechnol. 2017;35:1021–3. 29121022 10.1038/nbt.3958

[56] Jedwab A, Vears DF, Tse C, Gyngell C. Genetics experience impacts attitudes towards germline gene editing: a survey of over 1500 members of the public. J Hum Genet. 2020:1–11. 10.1038/s10038-020-0810-2 32737393

[57] Busch G, Ryan E, von Keyserlingk MAG, Weary DM. Citizen views on genome editing: effects of species and purpose. Agricult Hum Values. 2022;39:151–64.

[58] Hoffman‐Andrews L, Mazzoni R, Pacione M, Garland‐Thomson R, Ormond KE. Attitudes of people with inherited retinal conditions toward gene editing technology. Mol Genet Genom Med. 2019;7:e00803. 10.1002/mgg3.803 PMC6625087 31190471

[59] Neuhausser WM, Fouks Y, Lee SW, Macharia A, Hyun I, Adashi EY, et al. Acceptance of genetic editing and of whole genome sequencing of human embryos by patients with infertility before and after the onset of the COVID-19 pandemic. Reprod BioMed Online. 2023;47:157–63. 37127437 10.1016/j.rbmo.2023.03.013 PMC10330010

[60] van Dijke I, Lakeman P, Mathijssen IB, Goddijn M, Cornel MC, Henneman L. How will new genetic technologies, such as gene editing, change reproductive decision-making? Views of high-risk couples. Eur J Hum Genet. 2021;29:39–50. 32773775 10.1038/s41431-020-00706-8 PMC7852899

[61] Geuverink W, van El C, Cornel M, Lietaert Peerbolte BJ, Gitsels J, Martin L. Between desire and fear: a qualitative interview study exploring the perspectives of carriers of a genetic condition on human genome editing. Humanit Soc Sci Commun. 2023;10:1–9.

[62] Uchiyama M, Nagai A, Muto K. Survey on the perception of germline genome editing among the general public in Japan. J Hum Genet. 2018;63:745–8. 29545588 10.1038/s10038-018-0430-2 PMC6515154

[63] van Baalen S, Gouman J, Houtman D, Vijlbrief B, Riedijk S, Verhoef P. The DNA-dialogue: a broad societal dialogue about human germline genome editing in the Netherlands. CRISPR J. 2021;4:616–25. 34406039 10.1089/crispr.2021.0057

[64] Conley JM, Cadigan RJ, Davis AM, Juengst ET, Kuczynski K, Major R, et al. The promise and reality of public engagement in the governance of human genome editing research. Am J Bioeth. 2023;23:9–16. 37204137 10.1080/15265161.2023.2207502 PMC10367578

[65] O’Daniel JM, Ackerman S, Desrosiers LR, Rego S, Knight SJ, Mollison L, et al. Integration of stakeholder engagement from development to dissemination in genomic medicine research: approaches and outcomes from the CSER Consortium. Genet Med. 2022;24:1108–19. 35227608 10.1016/j.gim.2022.01.008 PMC9081226

[66] Boardman FK, Clark CC. What is a ‘serious’ genetic condition? The perceptions of people living with genetic conditions. Eur J Hum Genet. 2022;30:160–9. 34565797 10.1038/s41431-021-00962-2 PMC8821585

[67] Martani A. Changing the regulation of human germline genome editing: what does a truly broad societal debate entail? Law, Innov Technol. 2024;16:1–28.

[68] Houtman D, Geuverink W, Helmrich IRAR, Vijlbrief B, Cornel M, Riedijk S. “What if” should precede “whether” and “how” in the social conversation around human germline gene editing. J Community Genet. 2023;14:1–5. 10.1007/s12687-023-00652-0 PMC10444910 37326787

[69] Boëte C. Public engagement and communication: who is in charge? EMBO Rep. 2018;19:1–2. 29247079 10.15252/embr.201745379 PMC5757252

[70] ARRIGE Association for Responsible Research and Innovation in Genome Editing: ARRIGE; [Available from: https://www.arrige.org/.

