# Supplementary material for: A decade of public engagement regarding human germline gene editing: a systematic scoping review
Source: Eur J Hum Genet. 2024 Nov 28;33(5):570–9. doi: 10.1038/s41431-024-01740-6 (PMC12048525; doi:10.1038/s41431-024-01740-6)
Supplement: Supplementary file 1 — Supplementary Information [file 41431_2024_1740_MOESM1_ESM.docx]

**Supplementary Information on: *A decade of public engagement regarding human germline gene editing: a systematic scoping review***

Wendy P. Geuverink*, Diewertje Houtman*, Isabel R. A. Retel Helmrich, Joosje D. Kist, Lidewij Henneman, Martina C. Cornel, Sam R. Riedijk, and ‘The DNA dialogues’ Consortium

*These authors contributed equally

Contents

[‘The DNA dialogues’ Consortium 2](#_Toc180159762)

[Supplementary Information Text 1: Detailed description of search strategies (A&B) and databases searched 4](#_Toc180159763)

[Supplementary Information Table 1: Inclusion and exclusion criteria 11](#_Toc180159764)

[Supplementary Information Text 2: Sampling strategies to increase inclusivity, diversity and representativeness reported by the authors of included studies 12](#_Toc180159765)

[Supplementary Information Text 3: Reported limitations in inclusivity, diversity, and representativeness 13](#_Toc180159766)

[Supplementary Information Text 4: Reported output of public engagement 14](#_Toc180159767)

[Supplementary Information Text 5: Reported limitations that may affect the validity of the outputs 15](#_Toc180159768)

[Supplementary Information Text 6: Reported objectives of engagement practices beyond the scope of the study aimed at informing policy-making 16](#_Toc180159769)

[References 18](#_Toc180159770)

# ‘The DNA dialogues’ Consortium

| **Affiliation** | **Name consortium member** |
| --- | --- |
| Fontys University of Applied Sciences, the Netherlands | - D. Arets |
| Department of Clinical Genetics, Erasmus university Medical Center, Rotterdam, the Netherlands | - S.J.A.N. Arnold - B. Vijlbrief - D. Houtman - I.R.A. Retel Helmrich - J.D. Kist - S.R. Riedijk |
| Rathenau instituut, The Hague, the Netherlands | - S. van Baalen - F.H. van der Weij |
| Department of Legal Philosophy, Faculty of Law, Vrije Universiteit, Amsterdam, the Netherlands | - B. van Beers |
| NEMO KennisLink, Amsterdam, the Netherlands | - B. Burgers - E. Grob - J. Wiegertjes - M. van Woensel |
| Department of Human Genetics, Amsterdam University Medical Center, location Vrije Universiteit Amsterdam, Amsterdam, the Netherlands | - C.G. van El - W.P. Geuverink - L. Henneman - M.C. Cornel |
| Amsterdam Reproduction and Development Research Institute, Amsterdam, the Netherlands | - W.P. Geuverink - L. Henneman |
| Amsterdam Public Health Research Institute, Amsterdam, the Netherlands | - M.C. Cornel |
| GenomeScan, Leiden, the Netherlands | - D. Greeven |
| NPV – Zorg voor het leven, Veenendaal, the Netherlands | - Y. Geuze - van Horssen - E. van Hoek - Burgerhart |
| Rotterdam University of Applied Sciences, Willem de Kooning Academy, Rotterdam, the Netherlands | - M.D. Kasprzak |
| Erfocentrum (the Dutch National Information Centre on Heredity), the Netherlands | - J.W.G.A. Pot |
| Department of Genetics, Utrecht University Medical Center, Utrecht, the Netherlands | - T. Vrijenhoek |

Supplementary Information Text 1: Detailed description of search strategies (A&B) and databases searched

**A**: Search date: 2023/12/11

| **Database searched** | **Platform** | **Years of coverage** | **Records** | **Records after duplicates removed** |
| --- | --- | --- | --- | --- |
| Medline ALL | Ovid | 1946 - Present | 530 | 526 |
| Embase | Embase.com | 1971 - Present | 884 | 433 |
| Web of Science Core Collection* | Web of Knowledge | 1975 - Present | 440 | 110 |
| CINAHL* | EBSCO | 1982 - Present | 147 | 46 |
| PsycINFO | Ovid | 1806 - Present | 40 | 14 |
| Scopus | Scopus.com | 1823 - Present | 583 | 143 |
| International Bibliography of Social Sciences (IBSS) | ProQuest | 1951-present | 27 | 2 |
| The Philosopher’s Index | ProQuest | 1940-present | 53 | 10 |
| Hein online | heinonline.org |  | 54 | 21 |
| Additional Search Engines: Google Scholar** | | | 200 | 90 |
| **Total** | | | **2958** | **1395** |

*Science Citation Index Expanded (1975-present) ; Social Sciences Citation Index (1975-present) ; Arts & Humanities Citation Index (1975-present) ; Conference Proceedings Citation Index- Science (1990-present) ; Conference Proceedings Citation Index- Social Science & Humanities (1990-present) ; Emerging Sources Citation Index (2005-present).

**Google Scholar was searched via "Publish or Perish" to download the results in EndNote.

No other database limits were used than those specified in the search strategies.

*New references: 140*

**Medline**

(((Clustered Regularly Interspaced Short Palindromic Repeats/ OR Gene Targeting/ OR Genetic Engineering/ OR Cell Engineering/ OR Targeted Gene Repair/) AND (Germ-Line Mutation/ OR Germ Cells/)) OR (((germline* OR germ-line* OR germ-cell* OR germcell*) ADJ6 (editing OR modificat*)) OR ((heritab*) ADJ6 (gene OR genome*) ADJ6 (editing OR modificat*))).ab,ti.) AND (Social Values/ OR Public Opinion/ OR exp Attitude/ OR Culture/ OR Perception/ OR Morals/ OR exp Ethics/ OR Religion/ OR Conflict, Psychological/ OR Thinking/ OR Emotions/ OR "Interviews as Topic"/ OR "Surveys and Questionnaires"/ OR Delphi Technique / OR Community-Based Participatory Research / OR text mining/ OR Data Mining / OR (value* OR thought* OR feeling* OR emotion* OR sentiment* OR idea OR ideas OR stand OR reaction* OR conversation* OR deliberation* OR assembl* OR dialogue* OR engagement* OR interview* OR survey* OR focus-group* OR weighing OR argument* OR perspective* OR opinion* OR belief* OR believe* OR attitude* OR moral* OR perception* OR ethic* OR socioethic* OR bioethic* OR view OR views OR (position* ADJ3 statement*) OR relig* OR controvers* OR conflict* OR norm OR norms OR considerat* OR concern* OR (Citizen* ADJ3 (panel* OR assemb* OR science*)) OR Focusgroup* OR Questionnaire* OR (Deliberation ADJ3 event*) OR Delphi-stud* OR Delphi-technique* OR (Participator* ADJ3 research*) OR Polling OR Photovoice* OR Photo-voice* OR ((Text OR Opinion* OR data) ADJ mining) OR (Issue* ADJ3 mapping)).ab,ti,kw.) NOT ((exp animals/ OR exp plants/) NOT humans/) NOT (* Mitochondria / OR * Epigenomics / OR (somatic* OR mitochondr* OR epigenetic* OR epigenom*).ti.)

**Embase**

((('gene editing'/de OR 'crispr cas9 system'/de OR 'clustered regularly interspaced short palindromic repeat'/de OR 'gene targeting'/de OR 'genetic modification'/de OR 'genetic engineering'/de OR 'cell engineering'/de OR 'targeted gene repair'/de) AND ('germline mutation'/de OR 'germ line gene therapy'/de OR 'germ line'/de OR 'germ cell'/de)) OR 'germ line gene therapy'/de OR (((germline* OR germ-line* OR germ-cell* OR germcell*) NEAR/6 (editing OR modificat*)) OR ((heritab*) NEAR/6 (gene OR genome*) NEAR/6 (editing OR modificat*))):Ab,ti,kw) AND (value/de OR 'social value'/de OR argumentation/de OR perspective/de OR 'public opinion'/de OR attitude/exp OR belief/de OR beliefs/de OR perception/exp OR morality/exp OR ethics/exp OR religion/de OR conflict/de OR thinking/de OR emotion/de OR interview/de OR questionnaire/exp OR 'Delphi study'/de OR 'participatory research'/de OR 'text mining'/de OR 'data mining'/de OR (value* OR thought* OR feeling* OR emotion* OR sentiment* OR idea OR ideas OR stand OR reaction* OR conversation* OR deliberation* OR assembl* OR dialogue* OR engagement* OR interview* OR survey* OR focus-group* OR weighing OR argument* OR perspective* OR opinion* OR belief* OR believe* OR attitude* OR moral* OR perception* OR ethic* OR socioethic* OR bioethic* OR view OR views OR (position* NEAR/3 statement*) OR relig* OR controvers* OR conflict* OR norm OR norms OR considerat* OR concern* OR (Citizen* NEAR/3 (panel* OR assemb* OR science*)) OR Focusgroup* OR Questionnaire* OR (Deliberation NEAR/3 event*) OR Delphi-stud* OR Delphi-technique* OR (Participator* NEAR/3 research*) OR Polling OR Photovoice* OR Photo-voice* OR ((Text OR Opinion* OR data) NEXT/1 mining) OR (Issue* NEAR/3 mapping)):ab,ti,kw) NOT (([animals]/lim OR plant/exp) NOT [humans]/lim) NOT ('somatic mutation'/mj OR 'somatic cell'/mj OR mitochondrion/mj OR epigenetics/exp/mj OR (somatic* OR mitochondr* OR epigenetic* OR epigenom*):ti)

**Web of science**

TS=(((((germline* OR germ-line* OR germ-cell* OR germcell*) NEAR/5 (editing OR modificat*)) OR ((heritab*) NEAR/5 (gene OR genome*) NEAR/5 (editing OR modificat*)))) AND ((value* OR thought* OR feeling* OR emotion* OR sentiment* OR idea OR ideas OR stand OR reaction* OR conversation* OR deliberation* OR assembl* OR dialogue* OR engagement* OR interview* OR survey* OR focus-group* OR weighing OR argument* OR perspective* OR opinion* OR belief* OR believe* OR attitude* OR moral* OR perception* OR ethic* OR socioethic* OR bioethic* OR view OR views OR (position* NEAR/2 statement*) OR relig* OR controvers* OR conflict* OR norm OR norms OR considerat* OR concern* OR (Citizen* NEAR/2 (panel* OR assemb* OR science*)) OR Focusgroup* OR Questionnaire* OR (Deliberation NEAR/2 event*) OR Delphi-stud* OR Delphi-technique* OR (Participator* NEAR/2 research*) OR Polling OR Photovoice* OR Photo-voice* OR ((Text OR Opinion* OR data) NEAR/1 mining) OR (Issue* NEAR/2 mapping))) NOT ((animal* OR plant*) NOT human*)) NOT TI=((somatic* OR mitochondr* OR epigenetic* OR epigenom*))

**Scopus**

TITLE-ABS-KEY(((((germline* OR germ-line* OR germ-cell* OR germcell*) W/5 (editing OR modificat*)) OR ((heritab*) W/5 (gene OR genome*) W/5 (editing OR modificat*)))) AND ((value* OR thought* OR feeling* OR emotion* OR sentiment* OR idea OR ideas OR stand OR reaction* OR conversation* OR deliberation* OR assembl* OR dialogue* OR engagement* OR interview* OR survey* OR focus-group* OR weighing OR argument* OR perspective* OR opinion* OR belief* OR believe* OR attitude* OR moral* OR perception* OR ethic* OR socioethic* OR bioethic* OR view OR views OR (position* W/3 statement*) OR relig* OR controvers* OR conflict* OR norm OR norms OR considerat* OR concern* OR (Citizen* W/3 (panel* OR assemb* OR science*)) OR Focusgroup* OR Questionnaire* OR (Deliberation W/3 event*) OR Delphi-stud* OR Delphi-technique* OR (Participator* W/3 research*) OR Polling OR Photovoice* OR Photo-voice* OR ((Text OR Opinion* OR data) PRE/1 mining) OR (Issue* W/3 mapping))) AND NOT ((animal* OR plant*) AND NOT human*)) AND NOT TITLE((somatic* OR mitochondr* OR epigenetic* OR epigenom*))

**Cinahl**

(((MH Clustered Regularly Interspaced Short Palindromic Repeats OR MH Genetic Engineering) AND (MH Germ Cells)) OR TI(((germline* OR germ-line* OR germ-cell* OR germcell*) N5 (editing OR modificat*)) OR ((heritab*) N5 (gene OR genome*) N5 (editing OR modificat*))) OR AB(((germline* OR germ-line* OR germ-cell* OR germcell*) N5 (editing OR modificat*)) OR ((heritab*) N5 (gene OR genome*) N5 (editing OR modificat*)))) AND (MH Social Values OR MH Public Opinion OR MH Attitude+ OR MH Culture OR MH Perception OR MH Morals OR MH Ethics+ OR MH "Religion and Religions" OR MH "Conflict (Psychology)" OR TI(value* OR thought* OR feeling* OR emotion* OR sentiment* OR idea OR ideas OR stand OR reaction* OR conversation* OR deliberation* OR assembl* OR dialogue* OR engagement* OR interview* OR survey* OR focus-group* OR weighing OR argument* OR perspective* OR opinion* OR belief* OR believe* OR attitude* OR moral* OR perception* OR ethic* OR socioethic* OR bioethic* OR view OR views OR (position* N3 statement*) OR relig* OR controvers* OR conflict* OR norm OR norms OR considerat* OR concern* OR (Citizen* N3 (panel* OR assemb* OR science*)) OR Focusgroup* OR Questionnaire* OR (Deliberation N3 event*) OR Delphi-stud* OR Delphi-technique* OR (Participator* N3 research*) OR Polling OR Photovoice* OR Photo-voice* OR ((Text OR Opinion* OR data) N mining) OR (Issue* N3 mapping)) OR AB(value* OR thought* OR feeling* OR emotion* OR sentiment* OR idea OR ideas OR stand OR reaction* OR conversation* OR deliberation* OR assembl* OR dialogue* OR engagement* OR interview* OR survey* OR focus-group* OR weighing OR argument* OR perspective* OR opinion* OR belief* OR believe* OR attitude* OR moral* OR perception* OR ethic* OR socioethic* OR bioethic* OR view OR views OR (position* N3 statement*) OR relig* OR controvers* OR conflict* OR norm OR norms OR considerat* OR concern* OR (Citizen* N3 (panel* OR assemb* OR science*)) OR Focusgroup* OR Questionnaire* OR (Deliberation N3 event*) OR Delphi-stud* OR Delphi-technique* OR (Participator* N3 research*) OR Polling OR Photovoice* OR Photo-voice* OR ((Text OR Opinion* OR data) N mining) OR (Issue* N3 mapping)))) NOT ((MH animals+ OR MH plants+) NOT MH humans) NOT (MM Mitochondria + OR MM Epigenomics + OR TI(somatic* OR mitochondr* OR epigenetic* OR epigenom*))

**Psycinfo**

((((germline* OR germ-line* OR germ-cell* OR germcell*) ADJ6 (editing OR modificat*)) OR ((heritab*) ADJ6 (gene OR genome*) ADJ6 (editing OR modificat*))).ab,ti.) AND (Social Values/ OR Public Opinion/ OR exp Attitudes/ OR Perception/ OR Morality/ OR exp Ethics/ OR Religion/ OR Conflict/ OR (value* OR thought* OR feeling* OR emotion* OR sentiment* OR idea OR ideas OR stand OR reaction* OR conversation* OR deliberation* OR assembl* OR dialogue* OR engagement* OR interview* OR survey* OR focus-group* OR weighing OR argument* OR perspective* OR opinion* OR belief* OR believe* OR attitude* OR moral* OR perception* OR ethic* OR socioethic* OR bioethic* OR view OR views OR (position* ADJ3 statement*) OR relig* OR controvers* OR conflict* OR norm OR norms OR considerat* OR concern*).ab,ti.) NOT ((exp animals/ OR exp plants/) NOT humans/) NOT (* Mitochondria / OR * Epigenomics / OR (somatic* OR mitochondr* OR epigenetic* OR epigenom*).ti.)

**International Bibliography of the Social Sciences (IBSS)**

**The Philosopher’s Index**

(TI,AB(((germline* OR germ-line* OR germ-cell* OR germcell*) N/5 (editing OR modificat*)) OR ((heritab*) N5 (gene OR genome*) N/5 (editing OR modificat*)))) AND (AB,TI(value* OR thought* OR feeling* OR emotion* OR sentiment* OR idea OR ideas OR stand OR reaction* OR conversation* OR deliberation* OR assembl* OR dialogue* OR engagement* OR interview* OR survey* OR focus-group* OR weighing OR argument* OR perspective* OR opinion* OR belief* OR believe* OR attitude* OR moral* OR perception* OR ethic* OR socioethic* OR bioethic* OR view OR views OR (position* N/2 statement*) OR relig* OR controvers* OR conflict* OR norm OR norms OR considerat* OR concern*)) NOT (TI(somatic* OR mitochondr* OR epigenetic* OR epigenom*))

**Hein online**  (to download, make account and save to own folder)

(title:((germline* OR germ-line* OR germ-cell* OR germcell*) AND (editing OR modificat*)))

**Google scholar**

"germline|germcell editing|modification"|"germ line|cell editing|modification" value|values|weighing|argument|perspective|opinion|belief|believe|attitude|morality|perception|ethics|socioethics|bioethics|religion|controversy|conflict

'germline|germcell editing|modification'|'germ line|cell editing|modification' value|values|weighing|argument|perspective|opinion|belief|believe|attitude|morality|perception|ethics|socioethics|bioethics|religion|controversy|conflict

**Not in EndNote**

**Google**

"germline|germcell editing|modification"|"germ line|cell editing|modification" value|values|weighing|argument|perspective|opinion|belief|believe|attitude|morality|perception|ethics|socioethics|bioethics|religion|controversy|conflict file-type:pdf -pmid -doi

**JSTOR**

(germline OR germcell ) AND (editing OR modification) AND (value OR values OR weighing OR argument OR perspective OR opinion OR belief OR believe OR attitude OR morality OR perception OR ethics)

**B**: Search date: 2023/12/11

In this additional search **B** we conducted an Endnote full text search: germline* OR germ-line* OR germ-cell* OR germcell* OR (embryonic OR reproductive or heritable OR enhancement) to include articles that did not include these terms in the title or abstract.

| **Database searched** | **Platform** | **Years of coverage** | **Records** | **Records after duplicates removed** |
| --- | --- | --- | --- | --- |
| Medline ALL | Ovid | 1946 - Present | 665 | 660 |
| Embase | Embase.com | 1971 - Present | 723 | 353 |
| Web of Science Core Collection* | Web of Knowledge | 1975 - Present | 485 | 261 |
| CINAHL | EBSCO | 1982 - Present | 168 | 97 |
| PsycINFO | Ovid | 1806 - Present | 124 | 84 |
| Scopus | Scopus.com | 1823 - Present | 583 | 480 |
| International Bibliography of Social Sciences (IBSS) | ProQuest | 1951-present | 55 | 14 |
| The Philosopher’s Index | ProQuest | 1940-present | 23 | 12 |
| Additional Search Engines: Google Scholar** | | | 200 | 108 |
| **Total** | | | **3026** | **2069** |

*Science Citation Index Expanded (1975-present) ; Social Sciences Citation Index (1975-present) ; Arts & Humanities Citation Index (1975-present) ; Conference Proceedings Citation Index- Science (1990-present) ; Conference Proceedings Citation Index- Social Science & Humanities (1990-present) ; Emerging Sources Citation Index (2005-present).

**Google Scholar was searched via "Publish or Perish" to download the results in EndNote.

No other database limits were used than those specified in the search strategies.

*New references: 514*

1739

*In endnote search full text: germline* OR germ-line* OR germ-cell* OR germcell* OR (embryonic OR reproductive or heritable OR enhancement)*

**Medline**

(*Gene Targeting/ OR *Genetic Engineering/ OR *Targeted Gene Repair/ OR (((gene OR genome* OR genetic*) AND (editing OR modificat* OR engineering*))).ti.) AND (Social Values/ OR Public Opinion/ OR exp Attitude/ OR Perception/ OR Morals/ OR Social Media / OR (((global* OR public OR general OR population* OR consumer*) ADJ6 (view* OR opinion* OR perception* OR survey* OR questionnaire* OR belief* OR perception* OR attitud* OR accept* OR dialogue* OR discourse* OR think OR awareness* OR engag* OR understand* OR challenge* OR opportunit* OR reaction* OR reception* OR value*)) OR moralit* OR social-media*).ab,ti,kw. OR (general-public* OR opinion* OR belief* OR attitud* OR awareness* OR accept* OR dialogue* OR perception* OR values* OR value-based*).ti.) NOT ((exp animals/ OR exp plants/) NOT humans/) NOT (exp * neoplasms/ OR exp *sperm/ OR exp * agriculture/ OR (mouse OR mice OR rat OR rats OR murine OR agricultur* OR gmo* OR cancer* OR neoplas* OR sperm*).ti.)

**Embase**

('gene editing'/mj OR 'gene targeting'/mj OR 'genetic modification'/mj OR 'genetic engineering'/mj OR 'targeted gene repair'/mj OR (((gene OR genome* OR genetic*) AND (editing OR modificat* OR engineering*))):ti) AND ('public opinion'/de OR belief/de OR beliefs/de OR perception/exp OR morality/exp OR 'social media'/de OR (((global* OR public OR general OR population* OR consumer*) NEAR/6 (view* OR opinion* OR perception* OR survey* OR questionnaire* OR belief* OR perception* OR attitud* OR accept* OR dialogue* OR discourse* OR think OR awareness* OR engag* OR understand* OR challenge* OR opportunit* OR reaction* OR reception* OR value*)) OR moralit* OR social-media*):ab,ti,kw OR (general-public* OR opinion* OR belief* OR attitud* OR awareness* OR accept* OR dialogue* OR perception* OR values* OR value-based*):ti) NOT (([animals]/lim OR plant/exp) NOT [humans]/lim) NOT (neoplasm/exp/mj OR sperm/exp/mj OR agriculture/exp/mj OR (mouse OR mice OR rat OR rats OR murine OR agricultur* OR gmo* OR cancer* OR neoplas* OR sperm*):ti)

**Web of science**

TI=((((gene OR genome* OR genetic*) AND (editing OR modificat* OR engineering*)))) AND (TS=(((global* OR public OR general OR population* OR consumer*) NEAR/5 (view* OR opinion* OR perception* OR survey* OR questionnaire* OR belief* OR perception* OR attitud* OR accept* OR dialogue* OR discourse* OR think OR awareness* OR engag* OR understand* OR challenge* OR opportunit* OR reaction* OR reception* OR value*)) OR moralit* OR social-media*) OR TI=(general-public* OR opinion* OR belief* OR attitud* OR awareness* OR accept* OR dialogue* OR perception* OR values* OR value-based*)) NOT TI=((mouse OR mice OR rat OR rats OR murine OR agricultur* OR gmo* OR cancer* OR neoplas* OR sperm*))

**Scopus**

TITLE-ABS-KEY(((((germline* OR germ-line* OR germ-cell* OR germcell*) W/5 (editing OR modificat*)) OR ((heritab*) W/5 (gene OR genome*) W/5 (editing OR modificat*)))) AND ((value* OR thought* OR feeling* OR emotion* OR sentiment* OR idea OR ideas OR stand OR reaction* OR conversation* OR deliberation* OR assembl* OR dialogue* OR engagement* OR interview* OR survey* OR focus-group* OR weighing OR argument* OR perspective* OR opinion* OR belief* OR believe* OR attitude* OR moral* OR perception* OR ethic* OR socioethic* OR bioethic* OR view OR views OR (position* W/3 statement*) OR relig* OR controvers* OR conflict* OR norm OR norms OR considerat* OR concern* OR (Citizen* W/3 (panel* OR assemb* OR science*)) OR Focusgroup* OR Questionnaire* OR (Deliberation W/3 event*) OR Delphi-stud* OR Delphi-technique* OR (Participator* W/3 research*) OR Polling OR Photovoice* OR Photo-voice* OR ((Text OR Opinion* OR data) PRE/1 mining) OR (Issue* W/3 mapping))) AND NOT ((animal* OR plant*) AND NOT human*)) AND NOT TITLE((somatic* OR mitochondr* OR epigenetic* OR epigenom*))

**Cinahl**

(MM Genetic Engineering OR TI(((gene OR genome* OR genetic*) AND (editing OR modificat* OR engineering*)))) AND (MH Social Values OR MH Public Opinion OR MH Attitude+ OR Perception+ OR MH Perception OR MH Morals OR MH Social Media OR TI(((global* OR public OR general OR population* OR consumer*) N5 (view* OR opinion* OR perception* OR survey* OR questionnaire* OR belief* OR perception* OR attitud* OR accept* OR dialogue* OR discourse* OR think OR awareness* OR engag* OR understand* OR challenge* OR opportunit* OR reaction* OR reception* OR value*)) OR moralit* OR social-media*) OR AB(((global* OR public OR general OR population* OR consumer*) N5 (view* OR opinion* OR perception* OR survey* OR questionnaire* OR belief* OR perception* OR attitud* OR accept* OR dialogue* OR discourse* OR think OR awareness* OR engag* OR understand* OR challenge* OR opportunit* OR reaction* OR reception* OR value*)) OR moralit* OR social-media*) OR TI(general-public* OR opinion* OR belief* OR attitud* OR awareness* OR accept* OR dialogue* OR perception* OR values* OR value-based*)) NOT ((MH animals+ OR MH plants+) NOT MH humans+) NOT (MM neoplasms+ OR MM sperm+ OR MM agriculture+ OR TI(mouse OR mice OR rat OR rats OR murine OR agricultur* OR gmo* OR cancer* OR neoplas* OR sperm*))

**Psycinfo**

(*Gene Targeting/ OR *Genetic Engineering/ OR *Targeted Gene Repair/ OR (((gene OR genome* OR genetic*) AND (editing OR modificat* OR engineering*))).ti.) AND (Social Values/ OR Public Opinion/ OR exp Attitude/ OR Perception/ OR Morals/ OR Social Media / OR (((global* OR public OR general OR population* OR consumer*) ADJ6 (view* OR opinion* OR perception* OR survey* OR questionnaire* OR belief* OR perception* OR attitud* OR accept* OR dialogue* OR discourse* OR think OR awareness* OR engag* OR understand* OR challenge* OR opportunit* OR reaction* OR reception* OR value*)) OR moralit* OR social-media*).ab,ti. OR (general-public* OR opinion* OR belief* OR attitud* OR awareness* OR accept* OR dialogue* OR perception* OR values* OR value-based*).ti.) NOT ((animal.po.) NOT human.po.) NOT (exp * neoplasms/ OR exp *sperm/ OR exp * agriculture/ OR (mouse OR mice OR rat OR rats OR murine OR agricultur* OR gmo* OR cancer* OR neoplas* OR sperm*).ti.)

**International Bibliography of the Social Sciences (IBSS)**

**The Philosopher’s Index**

(TI(((gene OR genome* OR genetic*) AND (editing OR modificat* OR engineering*)))) AND (TI,AB(((global* OR public OR general OR population* OR consumer*) N/5 (view* OR opinion* OR perception* OR survey* OR questionnaire* OR belief* OR perception* OR attitud* OR accept* OR dialogue* OR discourse* OR think OR awareness* OR engag* OR understand* OR challenge* OR opportunit* OR reaction* OR reception* OR value*)) OR moralit* OR social-media*) OR TI(general-public* OR opinion* OR belief* OR attitud* OR awareness* OR accept* OR dialogue* OR perception* OR values* OR value-based*)) NOT TI(mouse OR mice OR rat OR rats OR murine OR agricultur* OR gmo* OR cancer* OR neoplas* OR sperm*)

**Google scholar**

"germline|germcell editing|modification"|"germ line|cell editing|modification" value|values|weighing|argument|perspective|opinion|belief|believe|attitude|morality|perception|ethics|socioethics|bioethics|religion|controversy|conflict

'germline|germcell editing|modification'|'germ line|cell editing|modification' value|values|weighing|argument|perspective|opinion|belief|believe|attitude|morality|perception|ethics|socioethics|bioethics|religion|controversy|conflict

**Not in EndNote**

**Google**

"germline|germcell editing|modification"|"germ line|cell editing|modification" value|values|weighing|argument|perspective|opinion|belief|believe|attitude|morality|perception|ethics|socioethics|bioethics|religion|controversy|conflict file-type:pdf -pmid -doi

**Hein online**

(title:(( gene OR genome* OR genetic*) AND (editing OR modificat*)))

**JSTOR**

(germline OR germcell ) AND (editing OR modification) AND (value OR values OR weighing OR argument OR perspective OR opinion OR belief OR believe OR attitude OR morality OR perception OR ethics)

# Supplementary Information Table 1: Inclusion and exclusion criteria

|  | **Inclusion** | **Exclusion** |
| --- | --- | --- |
| **Year of publication** | > 2011 | < 2012 |
| **Language** | English | Other than English |
| **Publication status** | Published  Peer reviewed  Full text available | Full text not available |
| **Type of publication** | Reports on engagement practices | Social media studies  Opinions/perspectives/ethical reflections  Animal studies  Books |
| **Topic** | Heritable/Human germline gene editing (intentional modification of the nuclear DNA of the germline i.e., embryo, zygote, gametes or precursor cells of gametes)  Human | (Somatic) gene therapy  Advanced therapy medicinal products (ATMPs)  Mitochondrial  Non-human |
| **Participants** | General public (including patients) | Experts  Scientists  Healthcare professionals  (Medical) students |

# Supplementary Information Text 2: Sampling strategies to increase inclusivity, diversity and representativeness reported by the authors of included studies

| Organizing tailored dialogues for specific target audiences including people with a migrant background or with lower literacy, school children, patients, youth, and elderly (35) |
| --- |
| Inviting both urban and semirural residents (34) |
| Recruitment in a village and a city in different parts of the country (33) |
| Recruitment through random sampling of residential addresses (20) |
| Selecting homes according to urban and rural proportionality (26) |
| Including participants from multiple countries in one study (8, 9, 15-17, 19) |
| Stratified sampling by, for instance, state, territory, gender (12) |
| Stratified sampling by gender, age, educational level, region, lifestyle and value measurements, and interactions between these characteristics (32) |
| Matching participants to a sampling frame based on gender, age, race, education, political ideology, political party identification, and political interest of the adult population (10, 22, 23) |
| Matching demographic characteristics to those of the adult population (18) |
| Selecting general public participants based on sex, age, and residential area according to the national census data (25) |
| Selecting participants to be as inclusive as possible based on demographics, such as race, gender, educational attainment, age, and religion/belief (21) |
| Sampling to ensure that the sex and age distribution of the respondents remained consistent with the demographics of the general population (28, 29) |
| Stratified purposive sampling to select a diverse sample based on demographics (1) |
| Stratified sampling to invite a broad range of participants based on attitudes toward prenatal intervention, age of the child with trisomy 21, and perceived burden of trisomy 21 on the child, having a living or deceased child with trisomy 18 or 13, educational background of the participant (3, 5) |
| Engaging those with the lived experience that only patients (and possibly their family members) possess (1-7, 25, 35) |

# Supplementary Information Text 3: Reported limitations in inclusivity, diversity, and representativeness

Despite the described efforts, 27 out of the 31 included studies (87%) reported limitations in the inclusivity, diversity, and representativeness (1-32).

The studies engaging the general public included more participants who are white (11, 16), from Western countries (16), open to the promises of new technology, highly educated and/or professionally exposed to genetics/genomics (in one study 96% identified themselves as student, professional or scientists) (11, 14, 16), higher literacy (28, 29), young (8, 11, 14), male (15), left leaning in political orientation (11) and liberal, educated, young, and urban (30). Eighteen studies (8-13, 15-29, 32) used online surveys and one study conducted an online focus group (31), thereby excluding people who do not have Internet access, for example in rural communities.

Several of the studies including patients and family of patients also indicated that participants’ representativeness of the targeted patient population was limited. For example, because participants were highly educated (1, 3-6), predominantly white or from Western origin (6, 7), recruited from one center only (4), members of a community/advocacy/condition-specific support group (1-3, 5) conditions were self-reported (25), only from a Christian religious background (7), financially secure (3, 5), self-selected (6, 7), were seeking fertility treatment or counselling for prenatal genetic testing (4, 6), or had chosen not to terminate a pregnancy affected by trisomy 21, 18 or 13 (3).

# Supplementary Information Text 4: Reported output of public engagement

In terms of output, 14 out of the 31 studies (45%) focussed on acceptance of, support for, or agreement with one or multiple potential applications of human germline gene editing (HGGE). In these studies, participants indicated to which degree they accepted, supported, or agreed with a certain HGGE scenario. The majority of these studies also investigated associations between acceptance and other variables including the COVID-19 pandemic (6), information exposure (22), attention to science fiction (23), demographic variables (8, 11), how information is presented (11), the impact of hereditary and moral concern (12), experiences with genetics or genomics (16), treatment characteristics (18), scientific knowledge (20), attitudes towards science (20), risk-benefit perceptions of HGGE (20), belief in human evolution (20), perceived likelihood of CRISPR uses (17), and patients versus public (25).

Nine of the 31 engagement studies (29%) yielded various outputs (2, 8, 9, 13, 15, 17, 21, 27, 32, 33). In these studies, the level of acceptance or support was complemented with more in-depth reasoning. Half of the studies (2, 27, 32, 33) yielded both qualitative and quantitative outputs. In one study, participants indicated their moral acceptance level on a pre- and post-video survey in combination with a more in-depth exploration of values through focus groups (2). Another study resulted in a typology of five perspectives that emerged from a combination between individual interview results and generalized survey findings (33). A third study looked at (changes in) opinions and acceptance rates in two Dutch national samples as well as among those who had been engaged in dialogue (32) . The latter group was also asked to self-report the impact of participation in a dialogue. Finally, one study used a survey that inquired about support for legalisation of HGGE and acceptance of transnational care which was complemented by qualitative data from the survey and from interviews (27).

# Supplementary Information Text 5: Reported limitations that may affect the validity of the outputs

Although all selected engagement practices aimed to capture the perspectives of participants, authors reported several limitations that may affect the validity of their outputs. These limitations included developments after the study had been performed that may have influenced participants’ perspectives, such as the news about the controversial HGGE experiment of He Jiankui and the decision by the US Supreme Court to reverse Roe v Wade, on the right to have an abortion (2, 6, 20, 22). Some authors also mentioned a potential change of attitudes as a result of more information, knowledge, (mis)understanding of the technologies, more engagement, or because the technologies are still (mostly) speculative which made it hard to elicit realistic potential perspectives that reflect actual decision-making of the participants (2-5, 8, 11, 24, 34). In the questionnaires used, HGGE in some cases was also framed in a way that could limit the validity and reliability of results: respondents were asked to assume that HGGE was safe and effective (16), not all characteristics of HGGE were included to limit complexity of the information provided (18), texts were kept short which may have led to uninformed answers (19), or examples of HGGE applications were not included which may have made it difficult to form an opinion (28, 29). Other limitations mentioned were the small effect sizes (22), not being able to investigate causality (23), not being able to identify duplicate attempts because of anonymity (16), excessive length of the questionnaire (35), the use of only or near only de-contextualized quantitative instruments (8) and therefore less in-depth understanding of why participants answered the way they did (6, 8, 13-15). In one interview study, the perspectives of the parents of children with disabilities, especially mothers, were included, while it has been documented that parents have different perspectives on treatment and cure than persons with the disability (3, 5). In two studies the authors mentioned that some participants may have been reluctant to express contrary opinions so as not to disrupt group cohesion or consensus or did not want to participate in heterogenous group discussions (21, 34).

# Supplementary Information Text 6: Reported objectives of engagement practices beyond the scope of the study aimed at informing policy-making

| “A final word on the value of surveys in this controversial territory. Public opinion cannot and should not tell us what is right to do. However, as the NAS report notes, “Public participation should be incorporated into the policy-making process for human genome editing and should include ongoing monitoring of public attitudes, informational deficits, and emerging concerns about issues surrounding enhancement".” (9) |
| --- |
| “Understanding the public’s attitudes is critical to education efforts and to formulating public policy.” (11) |
| “Understanding the influencing factors on public opinion toward gene editing is vital in guiding political policy and planning effective public education.” (15) |
| “If there is a groundswell of support for change, we would then call to action policymakers to review legislation which will allow scientists more freedom to investigate the potential of this technology to improve human health.” (14) |
| “As the discussions we observed here frequently paralleled the conversations by academic, industry, and government stakeholders on the ethical applications and limitations of gene editing, it is critical that the views of the public are not discounted, but rather occupy a central place in the democratic pursuit of scientific advancement.”(34) |
| “Users’ perspectives should be addressed, and they should be involved in shared governance and guiding further science and policy-making.” (4) |
| “Considering these attitudes, exploring beliefs and addressing concerns are vital research priorities to ensure science proceeds with GGE in an ethically and socially acceptable way. As with other technologies, the application of GGE is a strong determinant of individual attitudes. Thus, governing bodies ought to focus on applications rather than the technology itself to properly accommodate stakeholder perspectives.” (16) |
| “Involving citizens in the discourse about developments in technologies like CRISPR-Cas9 may have benefits for research processes, outcomes, and governance.” (33) |
| “From these discussions, a set of values was derived, that need to be protected in decision-making about further research and the possible introduction of the technology into clinical practice” (35) |
| “The reported public views can serve as input for future consideration of the ethics and governance of GGE.” (18) |
| “This research contributes to understanding public response to applications of genome editing, revealing differences that can help guide decisions related to adoption of these technologies.” (19) |
| “Keeping the goal of societal alignment in mind, dialogue may contribute to identifying the needs and values of the public since they become more aware of these themselves, leading to better informed decisions. At the same time, being able to place one’s own perspective in the context of others’ may contribute to more support for future decisions.” (32) |
| “Accordingly, there is not only a legal–ethical case as presented by Thaldar et al for not  obstructing research into and the eventual clinical trials of HHGE in South Africa, but also a  political case based on well informed, considered public opinion. This calls for urgent revision  of South African ethics guidelines that currently prohibit research on HHGE, and for dedicated HHGE legal regulations that provide a clear and comprehensive legal pathway for researchers who intend to conduct HHGE research and clinical trials.” (21) |
| “These findings provide evidence that attitudes towards gene editing are heterogeneous and public discourse, as well as policy making need to consider a range of arguments when evaluating this technology.” (24) |
| “Robust, open-framed public engagement is an essential component of this process. It can help both to elicit public preferences on whether and how HGE should proceed, and to develop and maintain a strong sense of public priorities in biomedical innovation funding and governance.” (31) |
| “The results of this study are relevant to the formulation of policies and regulations that will govern the introduction and application of human genome modification for gene therapy in Nigeria.” (30) |
| “For researchers and policy makers, the broad clinical application of gene-editing requires an established set of guidelines for when this kind of technology is appropriate for use.” (5) |
| "In line with Almeida and Ranisch’s (2022) conclusion that further consideration of different stakeholders’ views on human genome editing is crucial to translate society’s needs and values into thoughtful regulations and policies, this qualitative interview study aims to contribute to the global dialogue on the acceptability of human genome editing.” (7) |
| “Where does the public stand on the issue of human genome editing? And how do those attitudes translate into the desire for more public input on human genome editing as new applications emerge in the policy arena?” (10) |

# References

1. Hoffman‐Andrews L, Mazzoni R, Pacione M, Garland‐Thomson R, Ormond KE. Attitudes of people with inherited retinal conditions toward gene editing technology. Molecular genetics & genomic medicine. 2019;7(7):e00803.

2. Hollister BM, Gatter MC, Abdallah KE, Armsby AJ, Buscetta AJ, Byeon YJJ, et al. Perspectives of sickle cell disease stakeholders on heritable genome editing. The CRISPR journal. 2019;2(6):441-9.

3. Snure Beckman E, Deuitch N, Michie M, Allyse MA, Riggan KA, Ormond KE. Attitudes toward hypothetical uses of gene-editing technologies in parents of people with autosomal aneuploidies. The CRISPR Journal. 2019;2(5):324-30.

4. van Dijke I, Lakeman P, Mathijssen IB, Goddijn M, Cornel MC, Henneman L. How will new genetic technologies, such as gene editing, change reproductive decision-making? Views of high-risk couples. European Journal of Human Genetics. 2021;29(1):39-50.

5. Elliott K, Ahlawat N, Beckman ES, Ormond KE. “I wouldn't want anything that would change who he is.” The relationship between perceptions of identity and attitudes towards hypothetical gene-editing in parents of children with autosomal aneuploidies. SSM-Qualitative Research in Health. 2022;2:100151.

6. Neuhausser WM, Fouks Y, Lee SW, Macharia A, Hyun I, Adashi EY, et al. Acceptance of genetic editing and of whole genome sequencing of human embryos by patients with infertility before and after the onset of the COVID-19 pandemic. Reproductive BioMedicine Online. 2023;47(1):157-63.

7. Geuverink W, van El C, Cornel M, Lietaert Peerbolte BJ, Gitsels J, Martin L. Between desire and fear: a qualitative interview study exploring the perspectives of carriers of a genetic condition on human genome editing. humanities and social sciences communications. 2023;10(1):1-9.

8. McCaughey T, Sanfilippo PG, Gooden GE, Budden DM, Fan L, Fenwick E, et al. A global social media survey of attitudes to human genome editing. Cell stem cell. 2016;18(5):569-72.

9. Gaskell G, Bard I, Allansdottir A, Da Cunha RV, Eduard P, Hampel J, et al. Public views on gene editing and its uses. Nature biotechnology. 2017;35(11):1021-3.

10. Scheufele DA, Xenos MA, Howell EL, Rose KM, Brossard D, Hardy BW. US attitudes on human genome editing. Science. 2017;357(6351):553-4.

11. Weisberg SM, Badgio D, Chatterjee A. A CRISPR new world: attitudes in the public toward innovations in human genetic modification. Frontiers in public health. 2017;5:253896.

12. Critchley C, Nicol D, Bruce G, Walshe J, Treleaven T, Tuch B. Predicting public attitudes toward gene editing of germlines: the impact of moral and hereditary concern in human and animal applications. Frontiers in Genetics. 2019;9:704.

13. Hendriks S, Giesbertz NA, Bredenoord AL, Repping S. Reasons for being in favour of or against genome modification: a survey of the Dutch general public. Human reproduction open. 2018;2018(3):hoy008.

14. Treleaven T, Tuch BE. Australian public attitudes on gene editing of the human embryo. J Law Med. 2018;26(1):204-7.

15. McCaughey T, Budden DM, Sanfilippo PG, Gooden GE, Fan L, Fenwick E, et al. A need for better understanding is the major determinant for public perceptions of human gene editing. Human gene therapy. 2019;30(1):36-43.

16. Jedwab A, Vears DF, Tse C, Gyngell C. Genetics experience impacts attitudes towards germline gene editing: a survey of over 1500 members of the public. Journal of Human Genetics. 2020;65(12):1055-65.

17. So D, Sladek R, Joly Y. Assessing public opinions on the likelihood and permissibility of gene editing through construal level theory. New Genetics and Society. 2021;40(4):473-97.

18. Van Dijke I, van Wely M, Berkman B, Bredenoord A, Henneman L, Vliegenthart R, et al. Should germline genome editing be allowed? The effect of treatment characteristics on public acceptability. Human Reproduction. 2021;36(2):465-78.

19. Busch G, Ryan E, von Keyserlingk MA, Weary DM. Citizen views on genome editing: effects of species and purpose. Agriculture and Human Values. 2022;39(1):151-64.

20. Chen AA, Zhang X. Rethinking the knowledge-attitudes model and introducing belief in human evolution: examining antecedents of public acceptability of human gene editing. Health, Risk & Society. 2022;24(7-8):297-316.

21. Thaldar D, Shozi B, Steytler M, Hendry G, Botes M, Mnyandu N, et al. A deliberative public engagement study on heritable human genome editing among South Africans: Study results. Plos one. 2022;17(11):e0275372.

22. Howell EL, Kohl P, Scheufele DA, Clifford S, Shao A, Xenos MA, Brossard D. Enhanced threat or therapeutic benefit? Risk and benefit perceptions of human gene editing by purpose and heritability of edits. Journal of Risk Research. 2022;25(2):139-55.

23. Eichmeier AA, Bao L, Xenos MA, Brossard D, Scheufele DA. Fictional scenarios, real concerns: science fiction and perceptions of human genome editing. Journal of Science Communication. 2023;22(1):A08.

24. Halstead IN, Boehnke JR, Lewis GJ. Heterogeneous attitudinal profiles towards gene editing: Evidence from latent class analysis. Public Understanding of Science. 2023;32(2):159-74.

25. Uchiyama M, Nagai A, Muto K. Survey on the perception of germline genome editing among the general public in Japan. Journal of human genetics. 2018;63(6):745-8.

26. Macall DM, Madrigal-Pana J, Smyth SJ, Arias AG. Costa Rican consumer perceptions of gene-editing. Heliyon. 2023;9(8).

27. Kaur A. Could seeking human germline genome editing force journeys of transnational care? Multidisciplinary Journal of Gender Studies. 2020;9(2):184-209.

28. Akatsuka K, Hatta T, Sawai T, Fujita M. Genome editing of human embryos for research purposes: Japanese lay and expert attitudes. Frontiers in Genetics. 2023;14:1205067.

29. Sawai T, Hatta T, Akatsuka K, Fujita M. Human genome editing in clinical applications: Japanese lay and expert attitudes. Frontiers in Genetics. 2023;14:1205092.

30. Jibrilla M, Raji H, Okeke MI. Survey of attitude to human genome modification in Nigeria. Journal of Community Genetics. 2024;15(1):1-11.

31. Nelson JP, Tomblin DC, Barbera A, Smallwood M. The divide so wide: Public perspectives on the role of human genome editing in the US healthcare system. Public Understanding of Science. 2024;33(2):189-209.

32. Houtman D, Vijlbrief B, Polak M, Pot J, Verhoef P, Cornel M, Riedijk S. Changes in opinions about human germline gene editing as a result of the Dutch DNA-dialogue project. European Journal of Human Genetics. 2022:1-8.

33. Schuijff M, De Jong MD, Dijkstra AM. AQ methodology study on divergent perspectives on CRISPR-Cas9 in the Netherlands. BMC Medical Ethics. 2021;22(1):48.

34. Riggan KA, Sharp RR, Allyse M. Where will we draw the line? Public opinions of human gene editing. Qualitative Health Research. 2019;29(12):1823-35.

35. van Baalen S, Gouman J, Houtman D, Vijlbrief B, Riedijk S, Verhoef P. The DNA-dialogue: a broad societal dialogue about human germline genome editing in the Netherlands. The CRISPR Journal. 2021;4(4):616-25.
